# Feelings about birth by a group of high-risk pregnant women

**DOI:** 10.1590/0034-7167-2023-0059

**Published:** 2023-12-04

**Authors:** Juliana de Sousa de Almeida, Emily Marques Alves, Thelma Malagutti Sodré, Keli Regiane Tomeleri da Fonseca Pinto, Fabiana Fontana Medeiros, Catia Campaner Ferrari Bernardy

**Affiliations:** I Universidade Estadual de Londrina. Londrina, Paraná, Brazil

**Keywords:** Pregnancy, Pregnancy, High-Risk, Parturition, Sentiment Analysis, Humanization of Assistance, Embarazo, Embarazo de Alto Riesgo, Parto, Análisis de Sentimientos, Humanización de la Atención, Gravidez, Gravidez de Alto Risco, Nascimento, Análise de Sentimentos, Humanização da Assistência

## Abstract

**Objective:**

to understand feelings about birth among a group of high-risk pregnant women.

**Method:**

a descriptive and qualitative study, using Alfred Schütz’s social phenomenology as a philosophical theoretical framework. The study included 25 pregnant women undergoing high-risk prenatal care. The interview had the following guiding questions: tell me about your feelings regarding the moment of birth/childbirth; How do you deal with the high-risk diagnosis? What are your expectations for birth/childbirth?

**Results:**

five categories emerged: Fear of obstetric care; Fear of complications with the baby; Fear of cesarean section; Resilience in the face of high-risk pregnancy; and Expectations for birth.

**Considerations:**

high-risk pregnant women are afraid of the care they will receive, the risks and concern about the baby’s vitality at birth. The importance of care is emphasized, with a welcoming environment, bonding and communication between health team and pregnant woman.

## RESUMO

**Objetivo:**

compreender os sentimentos a respeito do nascimento por um grupo de gestantes de alto risco.

**Método:**

estudo descritivo e qualitativo, tendo a fenomenologia social de Alfred Schütz como referencial teórico filosófico. Participaram do estudo 25 gestantes em acompanhamento de pré-natal de alto risco. A entrevista contou com as seguintes questões norteadoras: fale-me sobre seus sentimentos em relação ao momento do nascimento/parto; Como você lida com o diagnóstico de alto risco? Quais suas expectativas para o nascimento/parto?

**Resultados:**

emergiram cinco categorias: *Medo da assistência obstétrica; Medo das complicações com o bebê; Medo da cesariana; A resiliência diante da gestação de alto risco*; e *Expectativas para o nascimento*.

**Considerações:**

as gestantes de alto risco sentem medo da assistência que receberão, dos riscos e apreensividade quanto à vitalidade do bebê no nascimento. Ressalta-se a importância de assistência com ambiente acolhedor, efetivação de vínculo e comunicação entre equipe de saúde e gestante.

## RESUMEN

**Objetivo:**

comprender los sentimientos sobre el parto de un grupo de gestantes de alto riesgo.

**Método:**

estudio descriptivo y cualitativo, utilizando como marco teórico filosófico la fenomenología social de Alfred Schütz. El estudio incluyó a 25 mujeres embarazadas que se sometían a atención prenatal de alto riesgo. La entrevista tuvo las siguientes preguntas orientadoras: cuénteme sobre sus sentimientos con respecto al momento del nacimiento/parto; ¿Cómo lidia con el diagnóstico de alto riesgo? ¿Cuáles son sus expectativas para el nacimiento/parto?

**Resultados:**

surgieron cinco categorías: Miedo a la atención obstétrica; Miedo a las complicaciones con el bebé; Miedo a la cesárea; Resiliencia ante el embarazo de alto riesgo; y Expectativas de nacimiento.

**Consideraciones:**

las gestantes de alto riesgo tienen miedo de la asistencia que recibirán, de los riesgos y aprensión en cuanto a la vitalidad del bebé al nacer. Se destaca la importancia de la asistencia con ambiente acogedor, vínculo y comunicación entre el equipo de salud y la gestante.

## INTRODUCTION

Childbirth is one of the most important moments in a woman’s life, because in addition to being a biological, social and cultural event, it is the moment when a mother is born. It is also the transition from a known reality to a new and often unknown one^(1)^.

Due to the intensification of interventionist and technical techniques, with time childbirth ceased to be a familiar, physiological moment and centered on woman care, becoming a medical practice, passing the leading role to the health team. Childbirth has often become distressing and traumatic, as pregnant women lose control of the situation and their autonomy, and it is up to doctors to conduct this process^(2-3)^.

The decision on the route of birth is rarely shared between doctor and pregnant woman. A study with high-risk pregnant women in the state of São Paulo shows that they had difficulties in choosing the route of childbirth, and those who did, mostly opted for vaginal childbirth, opposing the choice of cesarean section when the decision was made by the doctor. Women’s individual choice through childbirth did not represent, in this study, their role or autonomy. It is noteworthy the fact that less frequently women reported the possibility of joint decision between pregnant woman and doctor and that in 60.5% of these cases the cesarean section was the option of choice^(4)^. The route of childbirth should not be decided based only on technical knowledge, as it would become an unjustified omnipotence, therefore, it is necessary to listen to pregnant women and share the decision^(5)^.

Cesarean section is associated with increased risk of maternal and fetal morbidity^(6)^. The World Health Organization recommends that cesarean rates should be less than 15%, however data provided by the Information System on Live Births (SINASC - *Sistema de Informações sobre Nascidos Vivos*) show that in 2018 cesarean rates reached 55.9% of births in Brazil^(7)^. In Paraná, State Law 20.127/2020, which provides for the rights of pregnant women and women in labor, including the rights of pregnant women to opt for a cesarean section in elective situations, has considerably increased the rates of cesarean sections on request in the state, with unpublished data.

High-risk pregnancy is characterized by the presence of a higher risk of unfavorable development for women or baby, when compared to the average of the considered population. However, vaginal childbirth should be encouraged depending on the clinical conditions of both^(5)^.

There are a lot of expectations and feelings involved in birth, since the baby, before being born, is already imagined by women. In high-risk pregnancies, women often experience confusing and ambiguous feelings before childbirth. Concomitantly with the women’s desire to have their child, they also show concerns, fearing that the child will be born with problems or still be born dead. Even though they can imagine what their babies will be like and plan the birth, it often doesn’t happen the way they envisioned^(8)^.

In this context, understanding how women feel before childbirth is essential to help them reflect on the possibilities at the time of their child’s birth, stating that the pain is real, but it is bearable and does not need to be treated pharmacologically. It is believed that in this way there is relief from anxiety and increased security and autonomy over the way of conducting the childbirth. Moreover, it will improve women’s reproductive quality of life, reducing health care costs and negative repercussions on maternal and newborn health^(9)^.

Also, guiding pregnant women during prenatal care about childbirth methods and the possibilities of using non-pharmacological methods to relieve pain at work helps them to cope with their fears and insecurities, contributing to the reduction of unnecessary cesarean sections^(10)^.

To understand the feelings and anxieties that high-risk pregnant women experience regarding birth, we will use the phenomenological sociology of Alfred Schütz, because it provides a deepening of the meanings, intentions, beliefs and values of people, who attribute unique meanings to their actions and human relationships. The phenomenological perspective seeks to understand human beings and their worldview, with their true meanings and intentions, contemplating them as a unique being in their individuality that they experience and interpret the world in a personal way and interact with their peers, transforming them and being transformed^(11)^.

## OBJECTIVE

To understand feelings about birth among a group of high-risk pregnant women.

## METHOD

### Ethical aspects

The study followed regulatory norms for research with human beings, according to Resolution 466/2012 (BRASIL, 2012). The project was approved by the Research Ethics Committee of the *Universidade Estadual de Londrina* (UEL). Participants were instructed about the purpose of the research, and were only included after signing the Informed Consent Form.

### Study design and theoretical-methodological framework

This is a descriptive study with a qualitative approach, using Alfred Schütz’s social phenomenology as a philosophical theoretical framework, and the data were analyzed in light of it^(12)^. The research met the COnsolidated criteria for REporting Qualitative research (COREQ) items.

The social relationship is emphasized by Schütz as an essential element in the interpretation of subjective meanings implicit in the action of subjects in the world of life. Reality is the world of everyday life, also called the world of common sense or simply world of life^(12)^.

This framework is not interested in examining the individual particular behavior of each subject. The focus of interest is on what can be revealed as a standard characteristic or typical trait of an entire social group that is experiencing a given situation. This pattern is called “lived type”^(11)^. In this way, we will seek to understand the meanings in the intersubjective experience of the social relationship among pregnant women.

In phenomenology, it is men who give meaning to their actions. Social action is a human conduct intentionally designed by the subject, endowed with purpose. In this sense, understanding of the social turns to the behavior of individuals in relation to the motives that lead their action. For Schütz, the actions that relate to the achievement of future goals, expectations and projects are called “reasons for”. Actions that are based on the past, on the stock of available knowledge, on previous lived experience are “reasons why”^(11,13)^.

Faced with these concepts worked on by Schütz, it is intended to give voice to high-risk pregnant women, considering their subjectivities, singularities and intersubjective relationships, in an attempt to reveal the essence of the studied phenomenon, the feelings in relation to the birth revealed by “reasons why” and their expectations for this moment through “reasons for”.

### Data source and study setting

The study participants were twenty-five pregnant women who were undergoing prenatal care at the specialty outpatient clinic of a university hospital located in northern Paraná.

Pregnant women classified as high risk, who were in the second trimester of pregnancy, with chronological age above 18 years, guided in time and space were included. Pregnant women with fetal anomalies and primigravidae were excluded from the study. The delimitation of the number of participants occurred when the phenomena were unveiled and there was no more new information for analysis and discussion, i.e., the moment of theoretical saturation was considered when finding the response of the object under study^(14)^.

### Data collection and organization

Data were collected from June to September 2020, through a face-to-face interview with pregnant women carried out by the main researcher, at the outpatient clinic, in a private room. The researcher who carried out the collection has residency in obstetric nursing. Pregnant women were selected by convenience. They were invited to participate at the time of the post-consultation and, upon accepting, they were taken to this private environment. The interviews lasted an average of 20 minutes, were recorded on a cell phone and the speeches were transcribed in full. For data collection, three guiding questions were used: tell me about your feelings regarding the moment of birth/childbirth; How do you deal with the high-risk diagnosis? What are your expectations for birth/childbirth?

### Analysis

The results were analyzed manually and presented according to the methodological principles of phenomenological research in Schütz. Didactically. six interconnected principles are classified, not being presented in stages. The fourth principle is the subjective interpretation of the meaning of reasons “why” and “for” reported by participants, and the organization of these reasons is presented in categories, where speeches are interpreted^(15)^.

## RESULTS

A total of 25 pregnant women aged between 18 and 41 years were interviewed. Most were white (60%); were in a legalized civil union (53.3%); were housewives (66.6%); and many attended high school (86.6%), but not all completed it. There were no losses or refusals.

The mean number of pregnancies was three, but some women were multi-pregnant with seven pregnancies. Gestational age ranged between 20 and 38 weeks. The most prevalent maternal pathologies were hypertensive syndromes (43.3%), gestational diabetes (20%) and thyroid diseases (10%).

Childbirth is both a physiological phenomenon and a social experience. Thus, the woman develops her perceptions through the experience of pregnancy and childbirth, but also through the interrelationship among individuals. These interrelationships can result in positive or negative feelings. The reports presented below built the concrete categories of the experience of a group of pregnant women about their feelings about birth.

Women reported a feeling of fear related to some aspects, whether due to personal experiences or collective experiences, and expectations for humanized obstetric care. From this feeling and sensation, four categories related to the reasons why emerged: *Fear of obstetric care; Fear of complications with the baby; Fear of cesarean section*; and *Resilience in the face of high-risk pregnancy*, and a category related to the reasons for: *Expectations for birth*.

### Fear of obstetric care

*From the beginning I never wanted to have a normal childbirth, from the beginning. But lately I’ve been thinking about having a normal childbirth. I really wanted to have it humanized, I’m a little afraid of suffering obstetric violence, afraid of being alone.* (P2)

*Ah, we are very afraid, I’m afraid even though I’ve already had two childbirths. When I had my last child, I was a bit traumatized, it was kind of embarrassing. The doctor “harmed” a lot, he didn’t know how to treat us well and I got scared and I’m scared* [...]. (P4)

*I’m afraid of being mistreated and of something happening in the surgery. One reason ends up leading to another, because if you are treated well, you are not afraid, but if you arrive being mistreated, you are already afraid of what could happen.* (P9)

*My mother had the experience of losing my brother because of normal childbirth. He was born feet first and was curled up for a long time, he ended up dying, that’s why I have trauma*. (P10)

*I’m afraid of normal childbirth, because everyone says it hurts a lot. A friend of mine just had a baby and she said that she felt a lot of pain, a pain that has no explanation* [...]. (P15)

*I’m afraid of childbirth because my sister had a childbirth recently, and she said that they left her there alone during labor*. (P19)

### Fear of complications with the baby

*I already had a cesarean section where the baby was born and died. I had eclampsia gravidarum, my baby was not born well, he was hospitalized and died. This time I would like it to be different, I’m afraid it will happen again.* (P1)

*I’m afraid, quite a lot. When I stop to think about it, I’m very afraid of what might happen at the time. I’m afraid, and if it’s a cesarean section, afraid of the anesthesia they give, afraid of my babies being kept in an incubator.* (P5)

*Not really afraid of childbirth, I’m afraid of something happening to the baby. Not so much fear of pain, but of the baby not wanting to come out right away.* (P10)

*I’m afraid, seeing the reports we get a little scared of something happening to the baby.* (P25)

### Fear of cesarean section

*I’m not going to say that I’m not afraid, because I am. People with pregnancy are a foot in and out of the grave. It’s not fear of pain, because I already know it really hurts. It’s fear of not holding on* [...]. *They say that the second childbirth is faster. I’m afraid of the cesarean because of the anesthesia, because I’ve never had any anesthesia.* (P22)

*If it was a cesarean section, I would be more afraid because of the surgery, the cut, the recovery.* (P24)

*They said that maybe my childbirth will be a cesarean section, and I was scared to death. I’m afraid of the cesarean section because of the anesthesia, fear that it won’t work.* (P25)

*I’m a little afraid of the cesarean, my mother had a cesarean and, from what she reports, she felt a lot of pain afterwards, and I believe that the recovery is slower. It’s an extremely deep cut, it cuts through the uterus. I believe that a cesarean section is not a natural childbirth. When there is a risk in the mother’s or child’s life, they opt for a cesarean section, but if it’s not that, I don’t see a specific need for me*. (P6)

Some fears involving birth were present in this group of pregnant women, which motivated them to have an attitude of resilience, i.e., of facing and overcoming the adversities that generated their “reasons why” of fear. Therefore, when answering the question “How do you deal with the high-risk diagnosis”, they presented strategies and skills to overcome and resist unfavorable experiences, such as a high-risk pregnancy. The positive adaptation to obtain stability in the face of negative feelings was found in the distraction of these thoughts, in the search for information with the doctor in the face of the fear of the unknown, in the control of anxiety, in faith and religion, psychological help and use of integrative practices such as meditation.

### Resilience in the face of high-risk pregnancy

*I try not to get things in my head. I try to entertain myself with other things, like my work. It helps a lot not to think so much. The experiences of others end up affecting it a little, but I try not to get that in my head. I’m putting it in God’s hands, asking God to help me.* (P4)

*When I’m really in doubt, I ask my doctor. This thing of researching you end up seeing things you shouldn’t even see and thinking that this could happen to me and my babies.* (P5)

*I have a lot of faith in Blessed Virgin Mary, so I put faith first. I don’t really like researching, but I end up listening to co-workers talk about.* (P10)

*I try not to talk to a lot of people, and when I meet a pregnant woman, I don’t even ask much. I try to receive less opinion, stay calm, asking God a lot for it to work out regardless of what it is.* (P11)

*To control anxiety, I watch videos of moms showing after the baby is born.* (P15)

*My psychologist started seeing me now at the end of the pregnancy and both the psychologist and the psychiatrist will accompany me for a while, until after childbirth. I try to stay calm, but it’s hard.* (P20)

*I’m trying to practice more meditation, even because of high blood pressure, so I’m trying to prepare myself.* (P17)

Women expect humanized obstetric care at the time of birth, based on respect and care offered by professionals. The following category addressed the “reasons for”, which are the subject’s motives in order to achieve the future goals.

### Expectations for birth

It was noticed that there are expectations for a physiological vaginal childbirth or that the cesarean section does not have complications. They want cervical dilation to be quick during labor so that they do not have the suffering attributed to the pain of contractions. Pregnant women also expressed their desire for the presence of a companion as a form of support and family participation at the time of birth.

*I wanted it to be a very peaceful, quiet childbirth, with caring people to assist. People who can cheer us up, because when we are treated with stupidity, we are not so calm.* (P4)

*I would imagine that the perfect childbirth would be a normal childbirth, when they will come into the world at the right time* [...] *when they feel the desire to be born, without doing anything.* (P5)

*I want the experience to be good, with caring doctors. I would like to have the same experience as my sister, who had a baby a month ago and she really enjoyed motherhood. Good service helps a lot.* (P8)

*I would like everything to go well, God willing.* [...] *a good service is what we need the most, because I’m anxious. May they have more patience with us. I had two miscarriages and the doctors weren’t patient with me, I got depressed, with a lot of problems together* [...]. (P14)

*I want a normal childbirth, and if it is, I want my boyfriend to be there, for me to arrive at the maternity ward already having the baby, so as not to suffer too much. I prefer normal because it recovers faster. May doctors be considerate too.* (P19)

*I wanted this time to be peaceful, with my husband close to me all the time, and for me to be able to hear the baby’s cry. I don’t care about the pain; I just want him to be okay.* (P25)

## DISCUSSION

The vast majority of women reported fear associated with obstetric care during labor and childbirth. This fear was more frequent in pregnant women’s statements than the fear of labor pain. This insecurity may be related to the increase in unnecessary and often painful hospital interventions that pregnant women undergo, such as the use of synthetic oxytocin to speed up labor and the Kristeller maneuver, harmful practices that must be eliminated, and frequent vaginal examinations, the use of forceps or a vacuum extractor, and episiotomy, practices that are often used inappropriately^(6,16)^.

Although the World Health Organization’s recommendations for good childbirth care practices were carried out more than two decades ago, these procedures are still routinely used in many maternity hospitals, and when performed without the consent of patients, they are considered violations of women’s sexual and reproductive rights. Some women experienced these situations, and others heard reports about these experiences. This may have contributed to the reflection and perception that they do not want to suffer any type of obstetric violence during their childbirth experience.

The natural belief in the world, in its reality, the experience of the past and a probable future, and the fact that people are given to each other in a very similar way, constitute the philosophical foundation of the world of common sense. The person can act on it and on it and be modified by it, aware that their everyday world is not private but intersubjective, shared with other subjects, a social world, considered by Schütz^(12)^ as a scenario of social action, in which people enter into mutual relationship and try to understand each other.

Each explanation of the world is based on a stock of previous experiences, which can be one’s own immediate experiences as well as experiences passed on to them from their peers and above all their parents, teachers, and so on^(12)^. All these experiences are included in a form of knowledge baggage, which serves as a frame of reference to explain the world. All experiences in the world of life are brought into this framework. Therefore, objects and events in the world of life confront human beings from their typical characteristics. It is believed that the experiences lived by pregnant women influenced their feelings in relation to birth.

Humanization of obstetric care was desired by the interviewees. The literature^(17)^ addresses that it is only possible to make childbirth care humanized when pregnant women are heard and informed about their doubts, and then have autonomy to participate and decide on the procedures to be performed. On the contrary, when women have their sexual and reproductive rights denied, sequelae and emotional traumas such as fear and sadness may arise throughout their lives due to the unsuccessful experience of childbirth. It is also known that women feel more satisfied with the care they receive when professionals comply with humanized practices for childbirth and birth, prioritizing the minimum of obstetric interventions^(18-19)^.

Regarding the fear of labor pain, it was possible to identify in pregnant women’s speeches that there is a culture that continually relates the painful sensation to suffering and anguish. The fear of pain is a mental process that can frighten a woman throughout her pregnancy, even if she has never felt this pain at any time. However, in the parturition process, pain is physiological and natural, not being a sign that something is wrong, but a sign that natural physiology is happening and a new being is about to be born^(9)^.

For Schütz, planning is the anticipation of future events, and typification is part of anticipation. These anticipations are based on typical expectations in typical contexts. Typification refers to unique occurrence phenomena, and no person can register an experience without resorting to typification, therefore, they are individual and have social implications^(20)^.

In the world life of pregnant women, presenting expectations with childbirth typifies their actions in the gestation process, sometimes with the search for knowledge about childbirth, but if they are focused on difficulties and pain, such action can generate fear and insecurity.

The perception of pain during labor increases even more when medical interventions are performed, when women suffer obstetric violence or are left alone. Childbirth professionals must offer all women methods of pain relief, while helping them to build self-confidence during labor, maintaining a sense of mastery and well-being^(9)^.

In the speeches, popular knowledge about pain, transmitted collectively by family and friends, was observed as the origin of the fear of childbirth pain. This knowledge has a very important cultural value, and sometimes it determines women’s perception. Sociocultural knowledge is often able to strongly influence the construction of myths, beliefs and opinions that reflect on the personal experience of each one^(21)^.

Schütz explains that communication experiences with other people generate an interactive environment, and this environment is composed of events subjectively perceived differently by each person, but it allows understanding during communication to be mutual, as each person experiences their own situation and the situation experienced by the other person at that moment^(20)^. It is also in “face to face” situations that they can express their subjectivity and be assisted in their demands, but health professionals do not always interact effectively and adequately interpret their actions.

Another point observed in the present study was the fear associated with high-risk pregnancy and the baby’s vitality. High-risk pregnancy can cause obstetric and neonatal complications, with negative consequences for the mother or baby. Other studies corroborate the reports described here, as they show that feelings of fear and anxiety common in a high-risk pregnancy are intensified when there is a risk to mother/child dyad health. It becomes necessary for pregnant women to deal with their dreams and fantasies of an imaginary baby in the face of fear of losing it during childbirth or of not being born healthy^(22)^. As childbirth approaches, pregnant women’s expectations increase, being a period of anguish, assumptions and probabilities. This occurs because mothers are often not explained the real dimension of the risk they are subject to^(23)^.

In phenomenology, personal consciousness is continuous and mutable, which is why it is called “current of thought”, synonymously understood as “current of experiences or thoughts”, or even “current of conscious personal life”. These terms define the essence of each individual’s inner personal life. As people reflect on their experiences, the “intentionality” of consciousness occurs and, in this way, they become aware of something. The phenomenon of reflection can be interpreted in this study as the fear of complications with the baby and the deconstruction of a baby initially imagined by pregnant women^(20)^.

Some women in this study also reported fear of undergoing a cesarean section. It is known that cesarean section should be proposed exclusively in emergency conditions, since it is capable of causing an increased risk of prematurity and neonatal respiratory distress, when performed electively and without indication based on evidence. In addition to this, it is associated with the development of maternal morbidities, such as anesthetic and thromboembolic accidents, puerperal infections, hemorrhages and maternal death.

However, in high-risk pregnancies there are situations and clinical conditions in which normal childbirth is not possible and a cesarean section is necessary, often without prior planning. Since they experience a high-risk pregnancy, the route of childbirth is not known to pregnant women and they are often dependent on the medical decision at the time of hospitalization. Although many want to have a normal childbirth, they run the risk of being subjected to a cesarean section on medical advice. Women who undergo a cesarean section without prior planning may experience feelings such as fear, frustration, discouragement, insecurity and impotence^(24)^.

It is known that when experiencing pregnancy in her body, the woman becomes particularly more sensitive and emotional. It is anticipated that she will experience some degree of anxiety, stress, and sadness during the gestational period. However, it is observed in some women the intensification of anxiety symptoms and even the appearance of depressive conditions due to some stressful events. High-risk pregnancy and possible complications as well as fear of childbirth are stressful events considered risk factors for women’s psychological distress^(25)^.

Psychological distress can arise from the difficulty of adapting to new situations and adversities in life, and when this happens to pregnant women, there is a greater probability of complications in pregnancy, childbirth and the puerperium, which can have repercussions on mother-child dyad health, in addition to negative thoughts about motherhood and difficulties in caring for newborns^(25)^.

Resilience was unveiled in the face of unfavorable experiences with high-risk pregnancies, with strategies and skills to adapt to them, with emphasis on mental distraction maneuvers, search for information about childbirth and cesarean section, anxiety control and rescue of their faith and religion. Sometimes, some women did not know how to overcome the fear they experienced.

In the field of human and health sciences, understanding how people develop in the face of circumstances involves the definition of resilience, which is the ability that some individuals have to overcome the adversities of life^(26)^. It is essential for health professionals to help women develop resilience to adapt positively to new life situations.

Situations will be meaningful experiences when the present is lived reflectively, as the previous “now” moment is contained in today’s “now” in the form of “modification by retention”. If one goes through situations without reflection, one will only be within the flow of duration of that situation. The recovery of memories through memory is a requirement for rational construction, and what cannot be recovered can only be lived, never thought about, consequently, impossible to be verbalized. But the attitude assumed by people is called behavior, so they can fight the situation, suppress it or give in to it^(20)^.

One way of facing life’s difficulties is the exercise of spirituality, which in this study was used as a strategy to overcome the fear of birth. It is common to observe this behavior when coping with diseases, and spiritual care must be offered by professionals impartially to religion^(27-28)^.

Regarding expectations with birth, it was identified that this moment is not a neutral event, since it mobilizes high levels of anxiety, excitement and expectation for the meeting between the mother and the real baby, different from that imaginary and idealized during pregnancy. All study participants mentioned expectations involving good care, the presence of a companion, quick labor, healthy birth of babies and easy recovery. Furthermore, the experience with a previous childbirth influenced the preference for the next route of childbirth, and most stated that they preferred a normal childbirth. This finding agrees with a study found in the literature^(29)^ in which women who had a previous normal childbirth demonstrated a greater desire for a normal childbirth again.

Faced with the “reasons why” and “reasons for” based on the concepts of Alfred Schütz and the understanding of the concrete categories of the experience of a group of high-risk pregnant women, the experienced type “the fear of birth for high-risk pregnant women” is presented as the one who is afraid of childbirth and complications with the baby based on their previous experiences and also on other women close to them and on their collections of available knowledge, for various reasons, such as fear of not being able to give birth, fear of obstetric violence, fear of complications with the baby and fear of cesarean section.

In the experiences of an individual or a group in the social world, there is typification, something that typifies as a unique experienced structure and a pattern of behavior. The typification makes it possible to have an anonymous and objective knowledge of the analyzed situation, which manifests itself in the vivid description of social behavior, in the agreements of “reasons for” and “reasons why”^(13)^.

### Study limitations

The present study had as a limitation the sample belonging to a single high-risk outpatient service, and it was not possible to generalize the data to other services.

### Contributions to nursing, health, or public policies

Despite the application of this study in only one service, the results were significant and may represent the reality of other pregnant women and in other regions. These findings contribute to advances in obstetric care offered, especially by obstetric nurses, as they encourage reflection on high-risk pregnant women’s demands.

## FINAL CONSIDERATIONS

It was evident that high-risk pregnant women are afraid of the type of assistance they will receive and the pain of labor. They also feel insecure about the baby’s vitality at the time of birth and the risks of a possible cesarean section on medical advice; however, they seek to overcome these fears with information through their doctor, psychological professional help, control of anxiety through mental distraction and meditation, and through the faith in which they believe.

Humanized care is desired, based on respect and care offered by professionals as well as physiological vaginal childbirth or an uncomplicated cesarean section; quick and less painful dilation period; a healthy newborn; the easy post-childbirth recovery; the presence of a companion as a form of support; and the family’s participation at the time of their child’s birth.

The importance of interdisciplinary obstetric care is emphasized with the creation of a welcoming environment, bonding and communication between health team and pregnant woman, in addition to guidelines from the beginning of prenatal care to provide a greater level of satisfaction for them with the experience of gestating and giving birth.

It is hoped that with this study, there will be a greater understanding by health professionals regarding the fears of high-risk pregnant women related to childbirth, and in this way, help women to reframe their concepts during prenatal care as well as to develop a plan therapeutic since pregnancy.
